# Supplementary material for: The Risk Factors and Mechanisms of Azole Resistance of Candida tropicalis Blood Isolates in Thailand: A Retrospective Cohort Study
Source: J Fungi (Basel). 2022 Sep 20;8(10):983. doi: 10.3390/jof8100983 (PMC9604623; doi:10.3390/jof8100983)
Supplement: Supplementary file 1 [file jof-08-00983-s001.zip › jof-1855191-supplementary.pdf]

**Table S1.** Primers used in this study.

| Genes | Sequence (5'-3') | Reference |
|---|---|---|
| ERG11-1F | TCTGACATGGTGTGTGTGTG | [1] |
| ERG11-1R | ATTGATGCCATCAATGGCAG | [1] |
| ERG11-2F | ATCCCACAGGCTTATTTGAAA | [1] |
| ERG11-2R | GGTCTCTTTCCTTGGTTTTG | [1] |
| ERG11-3F | TGCTGAAGAAGCTTATACCC | [1] |
| ERG11-3R | CAAGGAATCAATCAAATCTCTC | [1] |
| ERG11-4F | TTACTGGAGACGTGACGCTG | This study |
| ERG11-4R | GGTGATCTGTGTCGGTTGGT | This study |
| ERG11-F for RT-PCR | CTACTCCCAAAAAAAACCATA | [2] |
| ERG11-R for RT-PCR | TAAACCTAATCCCAAGACATC | [2] |
| MDR1-F | TAAAGCAGGCTGGAGATGGA | [1] |
| MDR1-R | ACAACCTCCAACTATAGCTA | [1] |
| CDR1-F | TGAAGCCAGACCCGTAGTTG | [1] |
| CDR1-R | CCACTTTGCCCATCCTAACA | [1] |
| ACT-F | TTTACGCTGGTTTCTCCTTGCC | [1] |
| ACT-R | GCAGCTTCCAAACCTAAATCGG | [1] |

**Table S2.** Antifungal susceptibility test results, missense mutation of *ERG11* gene and gene expression levels of *Candida tropicalis* blood isolates.

| Isolate number | MIC (minimal inhibitory concentration, µg/ml) | | | | | | | | | | Missense mutations in *ERG11* gene | Fold expression level ($log_2$) | | |
|---|---|---|---|---|---|---|---|---|---|---|---|---|---|---|
| | FZ* | FZ** | IZ | VOR | PZ | FC | AND | MF | CAS | AB | | *ERG11* | *MDR1* | *CDR1* |
| **Fluconazole–resistant isolates** | | | | | | | | | | | | | | |
| 2 | 8 | 16 | 0.25 | 0.25 | 0.12 | <0.06 | 0.12 | 0.06 | 0.12 | 1 | ND | N/A | N/A | N/A |
| 4 | 128 | >256 | 4 | 0.5 | 0.5 | 0.12 | 0.12 | 0.03 | 0.12 | 1 | A395W, C461Y, T769C | 1.18 | 1.94 | 0.91 |
| 8 | >256 | >256 | >8 | 1 | 0.5 | <0.06 | 0.015 | 0.06 | 0.03 | 0.5 | A395T, C461T | 2.30 | 1.59 | 1.11 |
| 10 | >256 | >256 | >8 | 1 | 0.5 | <0.06 | 0.25 | 0.03 | 0.12 | 0.5 | A395T, C461T | 1.25 | 0.15 | 0.86 |
| 16 | >256 | >256 | >8 | 0.5 | 0.5 | <0.06 | 0.06 | 0.03 | 0.06 | 0.25 | A395T, C461T | 1.19 | 0.87 | 1.27 |
| 17 | 256 | 256 | >8 | 0.5 | 0.5 | <0.06 | 0.12 | 0.03 | 0.25 | 1 | A395T, C461T | 0.84 | 1.20 | 0.77 |
| 19 | >256 | >256 | >8 | 1 | 1 | <0.06 | 0.06 | 0.03 | 0.06 | 0.5 | A395T, C461T | 1.57 | 1.13 | 1.76 |
| 20 | 256 | >256 | >8 | 1 | 1 | <0.06 | 0.12 | 0.03 | 0.06 | 1 | A395T, C461T | 1.07 | 0.99 | 1.28 |
| 23 | >256 | 64 | >8 | 0.5 | 0.5 | <0.06 | 0.03 | <0.008 | 0.06 | 0.25 | A395T, C461T | N/A | N/A | N/A |
| 30 | >256 | >256 | >8 | 1 | 1 | <0.06 | 0.12 | 0.03 | 0.5 | 1 | A395T, C461T | 1.58 | 1.64 | 1.03 |
| 41 | >256 | 256 | >8 | 1 | 1 | <0.06 | 0.12 | 0.03 | 0.12 | 1 | A395T, C461T | 0.65 | 1.77 | 0.82 |
| 43 | 64 | 128 | 2 | 1 | 1 | <0.06 | 0.12 | 0.03 | 0.06 | 1 | A428G | 1.06 | 0.83 | 0.89 |
| 49 | 128 | 128 | 4 | 0.12 | 0.12 | <0.06 | 0.12 | 0.03 | 0.06 | 0.12 | A395T, C461T | 1.67 | 1.55 | 1.52 |
| 56 | >256 | >256 | >8 | 1 | 1 | <0.06 | 0.06 | 0.03 | 0.06 | 1 | A395T, C461T | 0.78 | 0.29 | 1.24 |
| 61 | 32 | 64 | 2 | 0.25 | 0.25 | 0.06 | 0.06 | 0.03 | 0.12 | 1 | A395W, C461Y | 1.21 | 0.78 | 0.85 |
| 64 | >256 | >256 | >8 | 1 | 1 | <0.06 | 0.12 | 0.03 | 0.06 | 1 | A395T, C461T | 0.98 | 0.38 | 0.62 |
| 67 | >256 | >256 | >8 | 2 | 2 | <0.06 | 0.06 | 0.03 | 0.06 | 0.5 | A395T, C461T | 0.72 | 0.61 | 0.78 |
| 68 | >256 | >256 | >8 | 1 | 1 | <0.06 | 0.06 | 0.03 | 0.06 | 1 | A395T, C461T | N/A | N/A | N/A |
| 78 | 64 | 64 | 1 | 1 | 1 | 0.12 | 0.12 | 0.06 | 0.06 | 1 | A428G | 0.92 | 0.84 | 1.16 |
| 84 | >256 | N/A | 4 | 0.5 | 0.25 | <0.06 | 0.12 | 0.06 | 0.25 | 2 | A395T, C461T | 1.43 | 0.68 | 2.27 |
| 89 | 8 | 16 | 0.25 | 0.25 | 0.12 | <0.06 | 0.12 | 0.06 | 0.12 | 0.25 | A395W, C461Y | 0.72 | 0.80 | 0.72 |
| 90 | 8 | 8 | 0.5 | 0.5 | 0.5 | 0.12 | 0.06 | 0.03 | 0.06 | 1 | ND | 1.35 | 1.84 | 1.13 |
| 99 | >256 | >256 | >8 | 1 | 1 | <0.06 | 0.06 | 0.03 | 0.06 | 1 | A395T, C461T | 2.22 | 0.49 | 0.73 |
| 105 | 128 | 128 | 8 | 0.5 | 0.5 | <0.06 | 0.12 | 0.06 | 0.06 | 1 | A395W, C461Y | 0.76 | 0.67 | 0.87 |
| 112 | 32 | 64 | 0.25 | 0.25 | 0.25 | <0.06 | 0.03 | 0.03 | 0.06 | 0.5 | A395W, C461Y | 1.25 | 1.33 | 0.44 |
| 116 | 64 | 64 | 2 | 0.25 | 0.12 | <0.06 | 0.25 | 0.06 | 0.12 | 1 | A395T, C461T | N/A | N/A | N/A |

| Isolate number | MIC (minimal inhibitory concentration, µg/ml) | | | | | | | | | | Missense mutations in *ERG11* gene | Fold expression level (log$_2$) | | |
|---|---|---|---|---|---|---|---|---|---|---|---|---|---|---|
| | FZ* | FZ** | IZ | VOR | PZ | FC | AND | MF | CAS | AB | | *ERG11* | *MDR1* | *CDR1* |
| 120 | 32 | 32 | 2 | 0.5 | 1 | <0.06 | 0.12 | 0.03 | 0.12 | <0.12 | ND | N/A | N/A | N/A |
| 121 | 256 | >256 | 8 | 1 | 1 | <0.06 | 0.06 | 0.03 | 0.06 | 1 | A395W, C461Y | 0.68 | 0.06 | 0.77 |
| 123 | >256 | >256 | >8 | 1 | 1 | <0.06 | 0.12 | 0.03 | 0.06 | 1 | A395T, C461T | 1.31 | 1.52 | 0.88 |
| 127 | >256 | >256 | >8 | 1 | 1 | <0.06 | 0.12 | 0.06 | 0.06 | 0.5 | A395T, C461T | 0.32 | 0.51 | 0.74 |
| 141 | >256 | >256 | >8 | 1 | 1 | <0.06 | 0.12 | 0.03 | 0.06 | 1 | A395T, C461T | 0.83 | 0.97 | 0.95 |
| 147 | >256 | >256 | >8 | 1 | 2 | <0.06 | 0.06 | 0.03 | 0.12 | 1 | A395T, C461T | 0.47 | 1.12 | 0.86 |
| 149 | >256 | >256 | >8 | 1 | 1 | <0.06 | 0.06 | 0.03 | 0.06 | 0.5 | A395T, C461T | 0.91 | 0.84 | 1.18 |
| 150 | >256 | >256 | >8 | 1 | 1 | <0.06 | 0.12 | 0.03 | 0.06 | 1 | A395T, C461T | 0.56 | 0.73 | 0.76 |
| 159 | 32 | 64 | 0.5 | 0.12 | 0.06 | <0.06 | 0.03 | 0.03 | 0.03 | 1 | A395T, C461T | 1.11 | 1.21 | 1.11 |
| 167 | 32 | 32 | 1 | 0.12 | 0.06 | <0.06 | 0.12 | 0.03 | 0.12 | 0.5 | ND | 0.88 | 0.56 | 0.68 |
| 170 | 32 | 64 | 2 | 0.5 | 0.5 | 0.12 | 0.12 | 0.03 | 0.06 | 0.5 | A395W, C461Y | 2.32 | 1.97 | 1.84 |
| 176 | 32 | 32 | 2 | 0.5 | 0.5 | 0.06 | 0.015 | 0.03 | 0.03 | 0.5 | ND | 0.12 | 0.70 | 1.27 |
| 178 | >256 | >256 | >8 | 1 | 1 | <0.06 | 1 | 2 | 8 | 1 | A395T, C461T | 0.81 | 0.73 | 0.74 |
| 181 | 256 | 256 | >8 | 1 | 1 | <0.06 | 0.12 | 0.03 | 0.12 | 1 | ND | 1.49 | 1.42 | 2.61 |
| 183 | 64 | 64 | 4 | 0.5 | 0.5 | <0.06 | 0.12 | 0.03 | 0.12 | 1 | A395W, C461Y | 0.92 | 1.63 | 0.78 |
| 185 | 32 | 32 | 1 | 0.25 | 0.12 | <0.06 | 0.12 | 0.015 | 0.06 | 0.12 | A395W, C461Y | N/A | N/A | N/A |
| 186 | 32 | 16 | 2 | 0.5 | 0.25 | <0.06 | 0.03 | 0.03 | 0.03 | 1 | A395W, C461Y | 1.68 | 3.12 | 2.53 |
| 197 | 32 | 32 | 4 | 0.25 | 0.12 | <0.06 | 0.12 | 0.03 | 0.12 | 1 | A395W, C461Y | 1.04 | 0.45 | 1.00 |
| 200 | >256 | 256 | >8 | 1 | 1 | <0.06 | 0.12 | 0.03 | 0.25 | 2 | A395T, C461T | 1.25 | 1.08 | 0.03 |
| 205 | 16 | 8 | 1 | 0.25 | 0.12 | <0.06 | 0.12 | 0.06 | 0.06 | 0.25 | ND | 0.71 | 0.59 | 0.82 |
| 213 | >256 | >256 | >8 | 1 | 0.5 | <0.06 | 0.12 | 0.03 | 0.06 | 1 | A395W, C461Y | 0.57 | 0.35 | 0.55 |
| 216 | 64 | 64 | 4 | 0.5 | 0.25 | <0.06 | 0.12 | 0.06 | 0.12 | 1 | A395W, C461Y | 1.29 | 0.88 | 1.32 |
| 225 | >256 | >256 | >8 | >16 | >8 | <0.06 | 0.12 | 0.06 | 0.06 | 0.5 | ND | 1.09 | 0.42 | 7.27 |
| 230 | 32 | 32 | 2 | 0.5 | 0.25 | <0.06 | 0.12 | 0.03 | 0.06 | 1 | A395W, C461Y | 1.66 | 0.53 | 1.20 |
| 232 | 8 | 16 | 1 | >16 | >8 | 0.12 | 0.06 | 0.03 | 0.06 | 1 | ND | 1.16 | 1.02 | 0.69 |
| 233 | >256 | >256 | >8 | 1 | 1 | <0.06 | 0.12 | 0.06 | 0.12 | 1 | A395T, C461T | 0.60 | 0.28 | 0.35 |
| 235 | >256 | >256 | >8 | 1 | 1 | <0.06 | 0.25 | 0.03 | 0.06 | 1 | A395T, C461T | 1.22 | 0.23 | 1.01 |
| **Fluconazole–susceptible isolates** | | | | | | | | | | | | | | |

| Isolate number | MIC (minimal inhibitory concentration, μg/ml) | | | | | | | | | | Missense mutations in *ERG11* gene | Fold expression level ($log_2$) | | |
|---|---|---|---|---|---|---|---|---|---|---|---|---|---|---|
| | FZ* | FZ** | IZ | VOR | PZ | FC | AND | MF | CAS | AB | | *ERG11* | *MDR1* | *CDR1* |
| 1 | 0.25 | 0.25 | 0.015 | 0.03 | 0.03 | <0.06 | 0.015 | 0.015 | 0.03 | 0.12 | ND | 1.48 | 1.56 | 1.85 |
| 5 | 1 | 0.25 | 0.12 | 0.25 | 0.25 | <0.06 | 0.06 | 0.03 | 0.06 | 1 | ND | 0.97 | 1.96 | 2.94 |
| 12 | 2 | 2 | 0.12 | 0.25 | 0.12 | <0.06 | 0.06 | 0.03 | 0.03 | 1 | ND | 1.23 | 1.01 | 2.54 |
| 13 | 2 | 0.25 | 0.12 | 0.12 | 0.12 | 0.25 | 0.12 | 0.03 | 0.06 | 0.5 | ND | 1.44 | 1.75 | 1.70 |
| 24 | 2 | 4 | 0.12 | 0.25 | 0.12 | <0.06 | 0.06 | 0.03 | 0.06 | 0.25 | ND | 1.03 | 1.41 | 0.91 |
| 29 | 1 | 0.25 | 0.06 | 0.03 | 0.015 | <0.06 | 0.015 | 0.03 | 0.03 | 0.25 | ND | 1.00 | 1.03 | 1.51 |
| 35 | 1 | 1 | 0.12 | 0.25 | 0.25 | 0.06 | 0.12 | 0.03 | 0.06 | 0.5 | ND | 1.16 | 0.96 | 0.88 |
| 38 | 2 | 2 | 0.12 | 0.25 | 0.25 | <0.06 | 0.03 | 0.015 | 0.06 | 1 | ND | 1.16 | 0.71 | 0.80 |
| 39 | 1 | 0.5 | 0.06 | 0.06 | 0.06 | <0.06 | 0.03 | 0.015 | 0.12 | 0.25 | ND | N/A | N/A | N/A |
| 48 | 2 | 2 | 0.12 | 0.03 | 0.03 | <0.06 | 0.25 | 0.12 | 0.12 | 0.5 | ND | 0.75 | 0.62 | 0.72 |
| 53 | 1 | 1 | 0.12 | 0.25 | 0.25 | <0.06 | 0.12 | 0.03 | 0.25 | 1 | ND | 0.78 | 0.72 | 1.01 |
| 55 | 1 | 0..5 | 0.06 | 0.25 | 0.12 | <0.06 | 0.12 | 0.015 | 0.25 | 0.5 | ND | 1.62 | 1.26 | 1.35 |
| 57 | 1 | 1 | 0.12 | 0.12 | 0.06 | <0.06 | 0.03 | 0.12 | 0.06 | 0.25 | ND | 1.05 | 2.64 | 1.19 |
| 72 | 2 | 2 | 0.12 | 0.25 | 0.12 | <0.06 | 0.12 | 0.03 | 0.12 | 0.5 | ND | 0.74 | 0.66 | 0.71 |
| 77 | 1 | 1 | 0.12 | 0.25 | 0.12 | <0.06 | 0.12 | 0.03 | 0.12 | 0.5 | ND | 1.14 | 1.16 | 1.08 |
| 92 | 2 | 2 | 0.12 | 0.5 | 0.25 | <0.06 | 0.03 | 0.03 | 0.06 | 1 | ND | 1.21 | 1.08 | 0.95 |
| 93 | 1 | 1 | 0.12 | 0.25 | 0.25 | <0.06 | 0.12 | 0.06 | 0.06 | 1 | ND | 0.90 | 0.74 | 0.60 |
| 102 | 1 | 1 | 0.06 | 0.12 | 0.06 | 64 | 0.03 | 0.03 | 0.06 | 0.5 | ND | 1.54 | 1.07 | 0.66 |
| 103 | 2 | 1 | 0.12 | 0.25 | 0.25 | <0.06 | 0.12 | 0.06 | 0.12 | 1 | ND | 0.95 | 0.85 | 0.90 |
| 108 | 1 | 0.5 | 0.03 | 0.06 | 0.06 | <0.06 | 0.03 | 0.015 | 0.06 | 0.25 | ND | 2.24 | 4.86 | 3.56 |
| 118 | 0.5 | 0.5 | 0.03 | 0.12 | 0.12 | <0.06 | 0.12 | 0.03 | 0.03 | 0.5 | ND | 1.20 | 0.50 | 0.66 |
| 119 | 1 | 1 | 0.12 | 0.12 | 0.06 | <0.06 | 0.06 | 0.03 | 0.03 | 0.5 | ND | 0.69 | 0.95 | 0.74 |
| 131 | 1 | 1 | 0.06 | 0.12 | 0.12 | <0.06 | 0.03 | 0.03 | 0.06 | 0.25 | ND | 1.53 | 1.48 | 1.16 |
| 132 | 1 | 1 | 0.06 | 0.12 | 0.25 | <0.06 | 0.06 | 0.06 | 0.12 | 0.5 | ND | 0.51 | 1.07 | 0.57 |
| 135 | 2 | 1 | 0.12 | 0.12 | 0.06 | <0.06 | 0.12 | 0.03 | 0.12 | 0.5 | ND | 0.94 | 0.81 | 0.76 |
| 136 | 1 | 1 | 0.12 | 0.25 | 0.12 | <0.06 | 0.06 | 0.03 | 0.06 | 1 | ND | N/A | N/A | N/A |
| 137 | 2 | 4 | 0.12 | 0.25 | 0.12 | <0.06 | 0.12 | 0.06 | 0.12 | 1 | ND | 1.29 | 1.21 | 0.95 |
| 140 | 2 | 2 | 0.12 | 0.25 | 0.12 | <0.06 | 0.03 | 0.03 | 0.06 | 0.25 | ND | 1.58 | 1.02 | 1.25 |

| Isolate number | MIC (minimal inhibitory concentration, μg/ml) | | | | | | | | | | Missense mutations in *ERG11* gene | Fold expression level ($log_2$) | | |
|---|---|---|---|---|---|---|---|---|---|---|---|---|---|---|
| | FZ* | FZ** | IZ | VOR | PZ | FC | AND | MF | CAS | AB | | *ERG11* | *MDR1* | *CDR1* |
| 189 | 2 | 1 | 0.12 | 0.25 | 0.12 | <0.06 | 0.12 | 0.03 | 0.25 | 1 | ND | 0.96 | 0.07 | 1.14 |
| 198 | 0.5 | 0.25 | 0.015 | 0.03 | 0.015 | <0.06 | 0.12 | 0.03 | 0.12 | 0.12 | ND | N/A | N/A | N/A |
| 208 | 1 | 1 | 0.06 | 0.06 | 0.06 | 0.06 | 0.12 | 0.03 | 0.12 | 0.5 | ND | 1.67 | 2.16 | 0.97 |
| 215 | 1 | 0.5 | 0.06 | 0.12 | 0.03 | 0.12 | 0.12 | 0.03 | 0.12 | 1 | ND | 0.46 | 0.64 | 0.27 |
| 221 | 2 | 1 | 0.12 | 0.25 | 0.12 | <0.06 | 0.06 | 0.03 | 0.06 | 0.5 | ND | 0.74 | 0.68 | 0.86 |
| 229 | 2 | 2 | 0.12 | 0.25 | 0.12 | 0.12 | 0.015 | 0.03 | 0.06 | 0.5 | ND | 0.60 | 0.83 | 0.59 |
| 239 | 2 | 2 | 0.12 | 0.25 | 0.25 | <0.06 | 0.06 | 0.03 | 0.03 | 1 | ND | 0.84 | 0.62 | 1.01 |
| 240 | 2 | 2 | 0.12 | 0.25 | 0.12 | <0.06 | 0.06 | 0.03 | 0.25 | 1 | ND | 0.55 | 1.00 | 0.68 |

*MIC value from Sentititre YeastOne, **MIC value from microbroth dilution along CLSI recommendation document M60.
Abbreviation: FZ, fluconazole; IZ, itraconazole; VOR, voriconazole; PZ, posaconazole; FC, 5-flucytosine; AND, anidulafungin; MF, micafungin; CAS; caspofungin; AB, amphotericin-B; N/A, not available; ND, not detected.

## References

1. Vandeputte, P.; Larcher, G.; Bergès, T.; Renier, G.; Chabasse, D.; Bouchara, J.-P. Mechanisms of azole resistance in a clinical isolate of Candida tropicalis. *Antimicrobial agents and chemotherapy* **2005**, *49*, 4608-4615, doi:10.1128/AAC.49.11.4608-4615.2005.
2. Jiang, C.; Dong, D.; Yu, B.; Cai, G.; Wang, X.; Ji, Y.; Peng, Y. Mechanisms of azole resistance in 52 clinical isolates of Candida tropicalis in China. *Journal of Antimicrobial Chemotherapy* **2012**, *68*, 778-785, doi:10.1093/jac/dks481.
